# CSP—A Model for In Vivo Presentation of *Plasmodium berghei* Sporozoite Antigens by Hepatocytes

**DOI:** 10.1371/journal.pone.0051875

**Published:** 2012-12-18

**Authors:** Saidou Balam, Jackeline F. Romero, Silayuv E. Bongfen, Philippe Guillaume, Giampietro Corradin

**Affiliations:** 1 Department of Biochemistry, University of Lausanne, Epalinges, Switzerland; 2 Ludwig Institute for Cancer Research, University of Lausanne, Epalinges, Switzerland; Museum National d’Histoire Naturelle, France

## Abstract

One target of protective immunity against the *Plasmodium* liver stage in BALB/c mice is represented by the circumsporozoite protein (CSP), and mainly involves its recognition by IFN-γ producing specific CD8+T-cells. In a previous *in vitro* study we showed that primary hepatocytes from BALB/c mice process *Plasmodium berghei* (*Pb*) CSP (*Pb*CSP) and present CSP-derived peptides to specific H-2k^d^ restricted CD8+T-cells with subsequent killing of the presenting cells. We now extend these observations to an *in vivo* infection model in which infected hepatocytes and antigen specific T-cell clones are transferred into recipient mice inducing protection from sporozoite (SPZ) challenge. In addition, using a similar protocol, we suggest the capacity of hepatocytes in priming of naïve T-cells to provide protection, as further confirmed by induction of protection after depletion of cross-presenting dendritic cells (DCs) by cytochrome c (cyt c) treatment or using traversal deficient parasites. Our results clearly show that hepatocytes present *Plasmodium* CSP to specific-primed CD8+T-cells, and could also prime naïve T-cells, leading to protection from infection. These results could contribute to a better understanding of liver stage immune response and design of malaria vaccines.

## Introduction

Immunization of rodents and humans with radiation- or genetically-attenuated sporozoites (SPZ) (RAS, GAS) confers pre-erythrocytic stage specific protective immunity to an infectious challenge [1], [2]. This protective immunity is mediated in part by CD8+ T-cells specific for the CSP and other not yet identified proteins [2]–[5]. Recently, it was demonstrated that both infected and sporozoite-traversed mouse primary hepatocytes can process the *Pb*CSP and present CSP-derived peptides to a specific H-2K^d^-restricted CD8+ T-cell clone *in vitro* but recognition of infected hepatocytes was the only relevant step in the elimination of infection [6]. Using bone marrow cell transfer into totally irradiated mice, it was also concluded that activation of protective CD8+ T-cell clones was due to antigen presentation by nonhematopoietic parenchymal cells [7]. Thus, the role of hepatocytes as antigen presenting cells (APCs) in the activation of primed T-cells to provide sterile protection seems to be accepted. Nevertheless, the role of liver cells and, in particular, hepatocytes in the activation of *Plasmodium*-specific CD8+ T-cells is not clearly elucidated. Chakravarty *et al.* [7] presented data supporting the role of lymph node dentritic cells (DCs) in presentation to parasite-specific T-cells while Leiriao *et al*. [8] supported the notion that apoptotic *Plasmodium*-infected hepatocytes provide antigen to liver DCs. While both pathways of antigen presentation co-exist, their role in providing a protective CD8+ T-cell response has not been established. In both studies, critical experiments such as the elimination of DCs and its consequence on sterile protection are missing. On the other hand, several publications point to the role of liver cells and hepatocytes in the mechanism of protection [2], [5], [9]–[11]. In addition, intravenous (iv) injection in mice of RAS gives rise to a more robust immune response and to sterile immunity compared to intradermal (id) immunization [12]. On the other hand, this difference in protection between iv and id injection is overcome by a higher dose of sporozoites [13]. Furthermore, immunization of mice with GAS is associated with a better immune response, probably due to the development of GAS liver stages and presentation of antigens other than CSP to specific T-cells [14].

It has been shown that CD8+ T-cells are primed by the DCs in the skin, and are then thought to exit the priming site and migrate to the liver where they can eliminate infection after recognizing antigens presented by hepatocytes [2], [7], [9], [15]. Thus, it was concluded that initiation of the CD8+ T-cell mediated immunity requires antigen presentation by only DCs [16]. On the other hand, the infected hepatocyte is the cell where SPZs develop and proliferate into the next liver forms. Furthermore, infection of hepatocytes is crucial for maintenance of anti- SPZ protective CD8+ T-cell response since protection was abrogated if hepatic stages were eliminated [2], [9], [15]. However, the ability of hepatocytes to prime naïve CD8+ T-cells and induce protective immunity remains unclear. It has also been illustrated that hepatocytes present CSP that is secreted directly into the cytosol, unlike DCs that cross-present *Plasmodium* antigens via endosomes [16]. These findings suggested that both in DCs and hepatocytes, the presentation of antigens requires the transporter associated with antigen processing (TAP) proteins [16]–[18] and that the cross-presenting DCs could be abrogated in mice after treatment with cytochrome c (cyt c) [16]. Determining if hepatocytes *in vivo* process and present *Plasmodium* antigens to naïve and primed T-cells may help in the rational identification of pre-erythrocytic vaccine candidates. To this purpose, we have established an *in vivo* protocol to address two questions: are hepatocytes capable of 1) *in vivo* stimulation of primed CD8+ T-cells and 2) priming naïve T-cells to protect mice against parasite challenge? We used different methods to address these two questions. First, we used intrasplenic (IS) transfer of *Pb*SPZ-infected hepatocytes from BALB/c mice into naïve, *TAP−/−* deficient mice (H-2K^b^ *)* and BALB/c mice (H-2K^d^) in the presence or absence of H-2K^d^-specific CD8+ T-cells (C7 clone) in order to determine the ability of primary hepatocytes to present antigen to primed CD8+ T-cells. Secondly, to show that hepatocytes could present antigen in the absence of dendritic cells, mice treated with cyt *c* were injected with irradiated SPZ (iSPZ) and challenged with live SPZ. Thirdly, mice were immunized with iSPZs deficient for the sporozoite microneme protein essential for cell traversal (*spect (−)* iSPZs), known for being incapable of cell traversal, but capable of infection and normal development [6], [19], [20] in order to show the role of infected hepatocytes in inducing a protective immune response. In all cases, mice were partially or totally protected from an infectious SPZ challenge as assessed by their level of blood parasitemia up to 14 days post infection.

## Materials and Methods

### Peptides

Peptides *Pb*CS_245–253_ (YIPSAEKI*)* and *Pb*CS_253–260_ modified with iodo-azidosalicylic acid (IASA) and azidobenzoic acid (ABA) groups, (*IASA*)-YIPSAEK(*ABA*)I representing the epitope for C7 clone (H-2K^d^-restricted and *Pb*CSP specific CTL) and S14 (H-2K^d^-restricted and an irrelevant CTL), respectively [3], [21], were synthesized by solid-phase F-moc chemistry. Peptide stock solutions (2 mg/ml) were prepared in PBS and stored at −20°C.

### Parasites

*Plasmodium berghei* ANKA wild-type (*wt*) and *spect (−)* SPZ were obtained after salivary gland dissection of infected female *Anopheles stephensi* mosquitos raised in the mosquito facility at the Department of Biochemistry, University of Lausanne, Switzerland as described previously [6], [20]. After dissection, salivary glands were homogenized in a glass grinder and released SPZ were counted and then diluted in sterile Dulbecco’s Modified Eagle Medium, DMEM (Gibco®, Life Technologies™, New York, NY).

### Animals

Six-to-12-week-old BALB/c (H-2K^d^) or *TAP−/−* (H-2K^b^) mice were obtained from Harlan Laboratories B.V. (Venray, Netherlands) or bred at the animal facility at the Department of Biochemistry (University of Lausanne, Switzerland). All mice were housed under pathogen-free conditions and handled according to the guidelines of the authorization N° 805.7 of the Service de la consommation et des affaires vétérinaires (Lausanne, Switzerland).

### Hepatocyte Isolation

SPZ-infected and naïve hepatocytes from BALB/c mice were obtained after collagenase perfusion of the liver as previously described [6], [20], [22]. Briefly, mice were sacrificed by CO_2_ inhalation, dissected, and the biggest lobule of liver was cut out. The lobule was perfused for 10 min with Ca^2+^-free HEPES (4-(2-hydroxyethyl)-1-piperazineethanesulfonic acid) buffer, pH 7.6 (Gibco® Invitrogen™, New York, NY) at 37°C at a rate of 5 ml/min. The lobule was then perfused with type IV collagenase (Sigma-Aldrich®, Steinheim, Germany) (HEPES buffer containing 0.04% type IV collagenase and 0.075% CaCl_2_-2H_2_O) for 5 min at 37°C. The perfused lobule was incubated for 10 min at 37°C in the collagenase solution. Using sterile pipettes, the tissue was gently teased apart to release cells and washed once with Ca^2+^-free HEPES buffer at 800 rpm for 30 s at 4°C. The pellet was gently re-suspended in DMEM, layered on 60% Percoll (GE Healthcare Bio-Sciences AB, Uppsala, Sweden) and centrifuged at 2000 rpm for 2 min at 4°C. The resulting pellet was re-suspended in complete culture medium (DMEM supplemented with 10% FCS, 1% penicillin streptomycin, 1% HEPES, and 0.05 mM of β-mecarptoethanol (β-ME, Sigma-Aldrich®), and centrifuged again at 800 rpm for 30 s at 4°C. The pellet was finally re-suspended again in 5 ml of DMEM for counting. Viability of isolated hepatocytes was assessed by light microscopy and trypan blue dye exclusion. As reported previously [6] hepatocyte contamination with Kupffer/dendritic cell markers is less than 1% as determined by FACS analysis [6], [10], [23], [24]. Then, mice were injected iv with 7×10^5^ live hepatocytes re-suspended in 200 µl of sterile DMEM. To obtain infected hepatocytes, 1×10^6^ of live or irradiated *wt PbSPZ* were injected iv through the tail vein of BALB/c mice. Two hours later, the liver was removed from the infected mouse and perfused as described above.

### Intrasplenic (IS) Transfer

Hepatocellular transplantation was carried out by direct injection of 7×10^5^ hepatocytes in 200 µl of sterile DMEM with a syringe into the splenic parenchyma of recipient mice that were anaesthetized with Isoflurane (Provet AG, Berne, Switzerland). Briefly, under aseptic and anaesthesia conditions, cautiously, with a chisel and a clip, the abdomen of the mouse was opened on the left flank. Using a clamp fitted, the spleen was gently pulled out and placed on a sterile piece of paper. The injection of cell suspension in the spleen parenchyma was carefully carried out at a rate of 10 µl per 10 respiratory cycles of the mouse. Suturing and clamping of the skin minimized leakage from the site of operation. Four hours later, 20 million *Pb*CSP-restricted CD8+ T-cells (C7-clone) or irrelevant CD8+ T-cells (clone S14) were injected iv through the tail vein in a volume of 500 µl of DMEM. Control mice received only hepatocytes.

### T-cell Clone Re-stimulation

The C7 and irrelevant S14 clones [3], [21] were re-stimulated weekly, maintained at 37°C and used as effector cells. Briefly, P815 cells (mastocytoma cells as antigen presenting cells, APC) were re-suspended (1×10^6^ cells/ml) in complete culture medium (DMEM supplemented with 10% FCS +1% of pyrimethamine-streptomycin +0.1% of β-ME and 1% of HEPES) and pulsed for 1 hour with *Pb*CSP-epitope peptides (1 µg/ml) specific for C7-and S14 clones. C7 and S14 were washed and re-suspended (2×10^6^/ml/well) in CTL culture medium (complete culture medium supplemented by 30 U/ml of mouse IL-2) in a 6-well plate. For re-stimulation, the respective clones were added to the 1×10^6^ pulsed and irradiated P815 cells (10000 rads/20 minutes) in the presence of 15×10^6^/well of irradiated-BALB/c spleen cells (5000 rads/10 minutes) in 6-well flat bottom plates. In the intrasplenic experiment, clones were re-stimulated and kept for two weeks in culture to make sure they were resting at the time of injection.

### Cytochrome c Treatment and Induction of CD8+ T-cells

BALB/c mice were depleted of cross-presenting DCs by iv treatment for 3 days with 15 mg/ml (5 mg/ml per day) of horse cyt c (Sigma-Aldrich®, St Louis, LA) in 100 µl of PBS (Gibco® Invitrogen™). Control mice received 100 µl of PBS alone. On the last day of treatment, mice were immunized iv with 1×10^5^ *wt Pb*iSPZ). Seven-to-ten days later, the frequency of both PE-conjugated SYIPSAEKI-tetramer and FITC-conjugated anti-mouse CD8 antibody (BD Biosciences, Allschwil, Switzerland) specific CD8+ T-cells was measured in different mouse tissues (blood = PBL, spleen, liver and lymph nodes (LN)) by flow cytometry.

### Real Time PCR (RT-PCR)

*In vivo* assessment of parasite loads was performed by RT-PCR as described previously [25]. Briefly, BALB/c mice were injected iv with 1×10^5^ *Pb*iSPZ (*wt* or *spect (−)*). Two hours later, total livers and spleens were isolated, perfused with PBS alone and total RNA was extracted. Then, cDNA was synthesized using specific primers for the *P. berghei* 18S rRNA (forward: 5′ AAGCATTAAATAAAGCGAATACATCCTTAC-3′ and reverse: 5′ GGAGATTGGTTTTGAC GTTTATGTG-3′) as described previously [25]. The DNA was thus amplified in the LightCycler 2.0 Instrument (Roche Diagnostics, Basel, Switzerland) using the program Roche LightCycler Run 5.32, and the relative parasite DNA load was thus determined in liver and spleen for each type of SPZ.

### Parasitemia Assessment

At different time points after the IS transfer or challenge with live *Pb*SPZs (iv), parasitemia was assessed by 10% Giemsa (Fluka®, Sigma-Aldrich®)-stained blood smears. Blood smear slides were air-dried and read by light microscopy (Olympus CH-2, Microscope Company, Hicksville, NY) to determine infected red blood cells (iRBC). Animals were protected against malaria if they remained negative in Giemsa-stained blood smears 2 weeks after receiving infected hepatocytes or live SPZ. Control animals were included to verify infectivity of SPZ or infected hepatocytes. In each control animal, parasitemia was detectable 7 days after IS transfer or challenge.

### Statistical Analysis

Different statistic tests were performed using GraphPad Prism software (version 6). Fisher’s exact test compares the proportion of mouse protection among various groups. The Mann-Whitney test was performed to compare parasite DNA load and frequency of *PbCSP*-specific CD8+ T-cells in PBL in two independent experiments or in different organs in the same experiment in which mice underwent different treatments (wild-type and Spect (−) iSPZ or PBS and cyt *c* groups). All p-values equal to or lower than 0.05 were considered significant.

## Results

### Infected Hepatocytes Present the *Pb*CSP-specific Epitope to Cloned CD8+ T-cells with Subsequent Protection against Malaria

To show that infected hepatocytes process and present *Pb*CSP, *TAP−/−* mice received BALB/c *Pb*SPZ-infected hepatocytes by IS transfer followed by iv injection of *Pb*CSP specific (clone C7) or irrelevant (S14 clone) CD8+ T-cells 4 h later. TAP −/− mice were selected to bypass the possibility that presentation could be performed by professional APCs through processing of apoptotic, infected hepatocytes or live SPZs possibly externally associated with BALB/c hepatocytes. In addition, the host vs graft immune response is minimized. All mice (8/8; 100%) that received infected hepatocytes and the specific C7 clone were protected from an infective sporozoite challenge **(** **Table 1** **, group A)**. In contrast, all mice receiving the irrelevant S14 clone were infected **(** **Table 1** **, group B**). In addition, 75% of naive *TAP−/−* mice treated with infected BALB/c hepatocytes were infected **(** **Table 1** **, group C).**

**Table 1:** Infected hepatocytes present a *Pb*CSP-specific epitope to primed CD8+ T-cells and protect mice against SPZ challenge.

	Treatment				
TAP−/− mouse group	BALB/c- SPZ infected hepatocytes	T cell clones	Mice protected/total injected	% of protection	*p-value*
**A**	yes	C7	8/8	100	*A versus B = 0.006*
**B**	yes	S14	0/3	0	*B versus C = 1*
**C**	yes	None	1/4	25	*A versus C = 0.018*

Each recipient *TAP−/−* mouse (H-2K^b^) received 7×10^5^ *Pb*SPZ-infected BALB/c (H-2k^d^) hepatocytes by IS transfer as described in Materials and Methods. Before IS injection, infected hepatocytes were isolated from BALB/c mice that were injected (iv) with 10^6 ^ANKA *wt Pb*SPZ 2 h earlier. C7 and S14 (20 million cells per mouse, iv) were injected into the corresponding group 4 h after IS transfer. Mice were protected if they remained parasite negative 2 weeks after infected hepatocyte transfer.

In order to show that the protective immune response is specific and not due to a bystander effect or a continuous secretion of cytokine by the C7 clone, *TAP−/−* mice were first infected with live *Pb*SPZ (1000/mouse in iv) or not. Ten hours later, mice received infected or naïve BALB/c hepatocytes together or without C7 clone as indicated in Table 2. Parasitemia determination of group C showed that only 1/7 (14%) of mice was protected **(** **Table 2** **, group C).** Other controls showed that protection occurred only in the group that received infected BALB/c hepatocyte and C7 clone (**Table 2** **,** **group A)**.

**Table 2:** Specificity of activation of C7 clone and protection**.**

	Treatment					
TAP−/− mouse group	Anka *wt* SPZ	BALB/c hepatocytes	C7 clone	Mice protected/total injected	% of protection	
**A**	None	SPZ infected	yes	5/5	100	***p-value***
**B**	None	SPZ infected	none	0/5	0	*A versus B = 0.008*
**C**	Yes	Naïve	yes	1/7	14	*A versus C = 0.015*
**D**	Yes	none	yes	0/3	0	*A versus D = 0.018*
**E**	Yes	none	none	0/3	0	*A versus E = 0.018*

Mice were first injected (iv) or not with 10^3^ live wild-type (*wt*) ANKA *Pb*SPZ 10 h before they received or not 7×10^5^ naïve-BALB/c hepatocytes and C7 clone as indicated above. Infected hepatocytes were isolated from BALB/c mice injected with 10^6^ ANKA *wt Pb*SPZ 2 h earlier; and naïve BALB/c hepatocytes were isolated from naïve BALB/c mice. Mice were considered protected if they remained parasite negative 2 weeks after infection.

### Infected Hepatocytes Prime *Pb*CSP-specific CD8+ T-cells and Protect Mice against SPZ Challenge

Since the infected hepatocytes can reactivate resting CSP-specific CD8+ T-cells and induce protective immunity **(** **Table 1** **and** **2** **)**, the next step was to determine if they could also prime naïve T-cells to protect mice against live SPZ challenge. To this effect, we injected iSPZ-infected BALB/c hepatocytes into naive BALB/c mice before challenging with live SPZ. Considering that the immunization procedure may give rise to a sub-optimal immunity (about 700 infected hepatocytes injected IS if the overall infection efficacy is estimated to be 10%), mice were challenged with a sub-optimal or optimal dose of live SPZ (2×10^3^ and 5×10^3^) that led to 60% and 100% infection in naïve mice, respectively, with an overall protection of 13% **(** **Table 3** **).** In contrast, mice receiving iSPZ-infected hepatocytes were protected at 64% (7/11; *p = 0.014*) **(** **Table 3** **),** suggesting that the infected hepatocytes could contribute to the priming of naïve T-cells and protection of mice against infection**.**

**Table 3:** BALB/c mice injected with iSPZ-loaded hepatocytes are protected against SPZ challenge.

		2 weeks after challenge		
Mouse group (n of SPZ for challenge)	Treatment	Mice protected/total challenged	% of protection	*p-value: IS versus naive*
**A (**2000)	IS	5/5	100	***0.167***
	Naive	2/5	40	
**B (**5000)	IS	2/6	33	***0.125***
	Naive	0/10	0	
**Total**	**IS**	**7/11**	**64**	***0.014***
	**Naive**	**2/15**	**13**	

BALB/c mice were injected (IS transfer) with 7×10^5^ *Pb*iSPZ-infected or naïve BALB/c hepatocytes. Infected hepatocytes were obtained from BALB/c mice immunized with 10^6^ ANKA *wt* iSPZ 2 h earlier. Recipient mice were then challenged with two different doses (2×10^3^ and 5×10^3^, respectively, A and B) of live *Pb*SPZ one week later. Mice were considered protected if they remained parasite negative 2 weeks after challenge.

**Figure 1:**
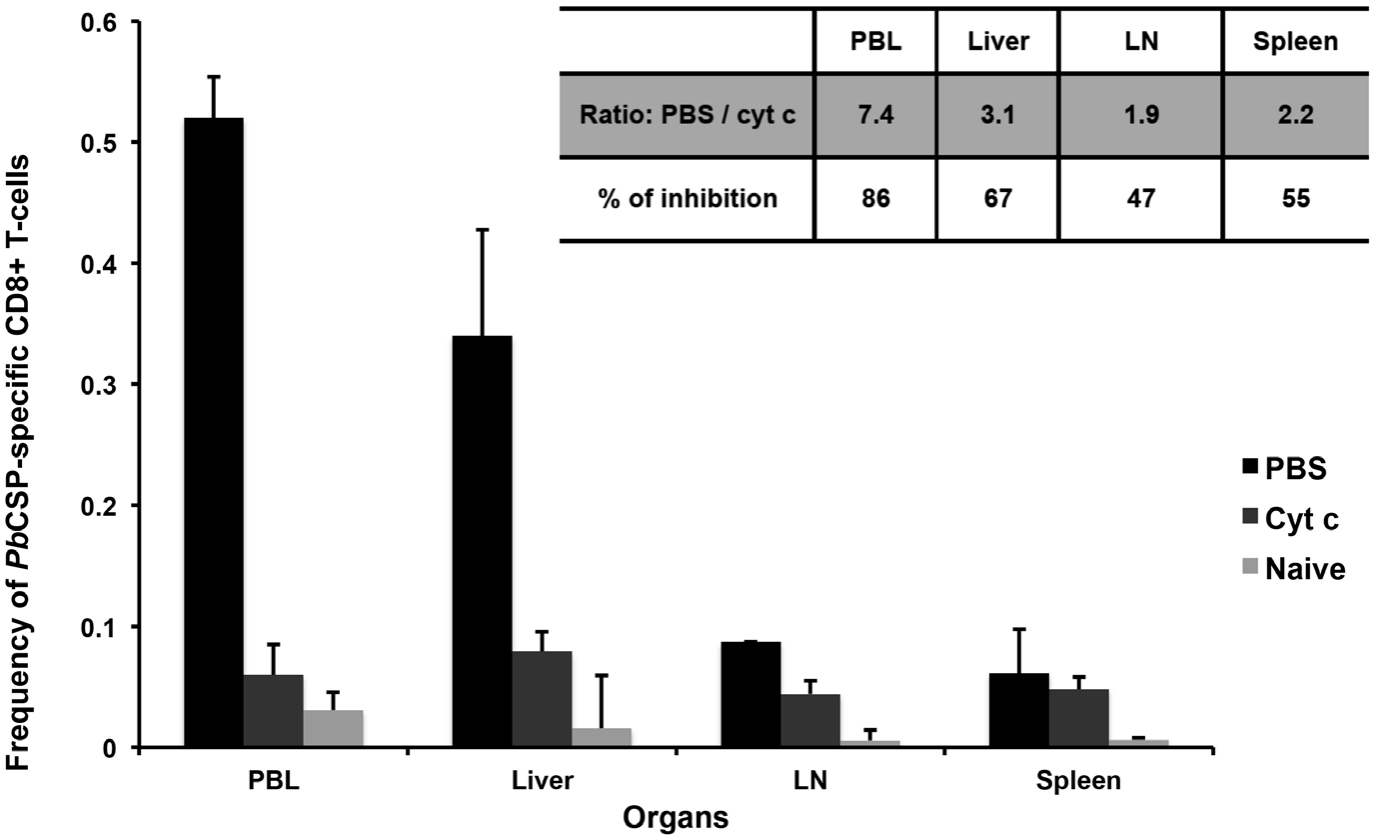
Level of *Pb*CSP-specific CD8+ T-cells in different organs of BALB/C mice after treatment with either cyt c or PBS and immunization with *Pb*iSPZ. Two groups of BALB/c mouse were pre-treated (iv through the tail vein) with 5 mg/mouse of cyt c per day for 3 days in 100 µl of PBS or with 100 µl of PBS alone. Immediately after the last treatment, mice were immunized with 1×10^5^ *Pb*iSPZ in 100 µl of RPMI. One-week later, *Pb*CSP epitope-specific CD8+ T-cell frequency (2 mice per group of treatment) was evaluated in peripheral blood lymphocytes (PBL), liver, lymph nodes (LN) and spleen. Naïve (receiving neither cyt c, PBS nor iSPZ) mice were used as negative control. Inserted panel shows the fold change in frequency and the percentage of inhibition of *Pb*CSP-specific CD8+ T-cells in cyt c-treated compared to PBS alone-treated groups.

To further corroborate that hepatocytes could prime naïve T-cells and induce protection, BALB/c mice were treated with cyt c to delete cross-presenting DCs before and during iSPZ immunization. This protocol was established according to previous studies which showed that cross-presenting DCs can be largely depleted after *in vivo* cyt c treatment [16], [26]. The first experiment (as pilot) showed a significant reduction of 60% of *Pb*CSP_245–253_ specific CD8+ T-cells in PBL after cyt c treatment (data not shown). In the second experiment, the analysis was extended to other organs (LN, spleen and liver) (**Fig. 1** **).** Thus, FACS analysis showed that induction of *PbCSP*-specific CD8+ T-cells was highly reduced in the cyt c- compared to the PBS -treated mice in PBL and liver (86% and 67%, respectively), while this reduction was about 50% in LN and spleen **(** **Fig. 1**, **inserted panel**). Taking the PBL data from the two experiments or from all organs in the second experiment, normalizing and combining them, we obtained p values of 0.003 (PBL) and 0.001 (organs) for the cyt c-treated compared to PBS groups. In spite of these differences, both groups were protected after SPZ challenge **(** **Table 4** **).**

**Table 4:** Protection from infection in cyt c- and PBS- treated mice.

Treatment	Mice protected/total challenged	% of protection
**Cyt c**	10/10	**100**
**PBS**	10/10	**100**
**Naive**	1/10	**10**

Protection of mice after cyt c or PBS treatment from live SPZ challenge of two independent experiments (5 mice per group in each experiment). Mice were either pre-treated iv for 3 days with 5 mg/mouse of horse cyt c per day in 100 µl of PBS or with 100 µl of PBS alone. On the last day of treatment, mice were immunized iv with 1×10^5^ *Pb*iSPZ in 100 µl of RPMI. One week later, mice were challenged with 5×10^3^ live *Pb*SPZ. Parasitemia was checked at 1 and 2 weeks after challenge. Mice were considered protected if they remained parasite negative 2 weeks after challenge.

### Protective Immune Response Induced in BALB/c Mice Immunized with *spect (−)* iSPZ

**Figure 2:**
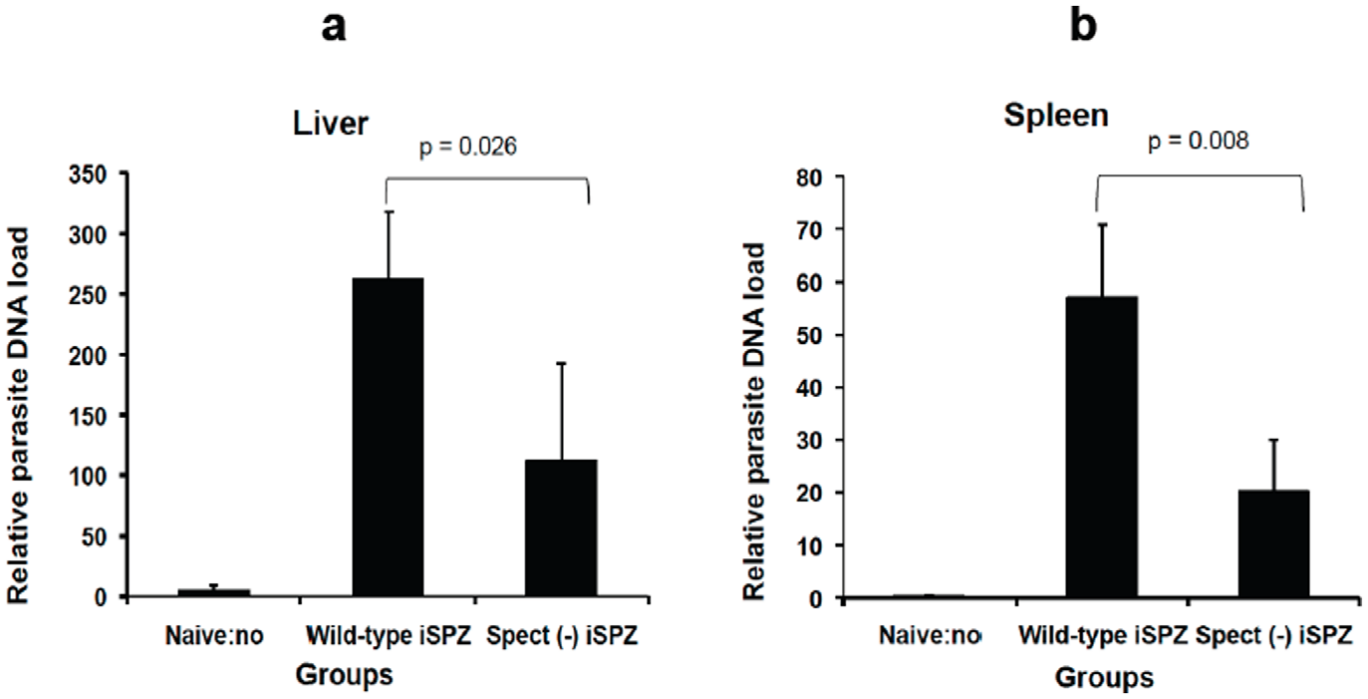
Relative comparative *wt* and *spect (*−*)* parasite DNA load in spleen and liver of immunized mice. To compare relative load of *wt* and *spect (*−*)* parasite DNA in liver and spleen, BALB/c (3 mice per group) were immunized iv on tail with 1×10^5^ iSPZ (*wt* or *spect (*−*)*) in 500 µl of RPMI. Two hours later, a real-time PCR was performed following extraction of respective parasite RNA. Every sample was done in duplicate. To avoid any contamination by eventual parasite from blood, the liver and spleen were perfused with PBS before performing the RT-PCR. Figures **a** and **b** represent *wt* and *spect (*−*)* parasite DNA load, respectively, in the liver and spleen 2 h after iSPZ injection (iv). Naïve mice (receiving only 500 µl iv of DMEM) were used as a control.

**Figure 3:**
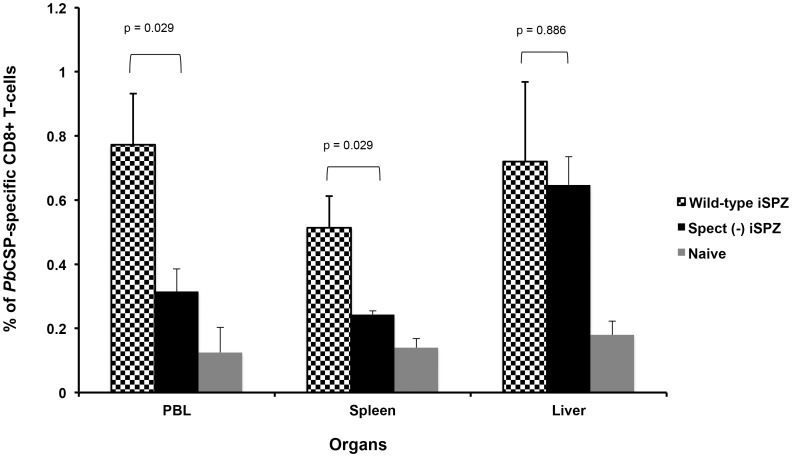
Frequency of *Pb*CSP-specific CD8+ T-cells in different organs of BALB/c mice immunized with *wt* or *spect (*−*)* iSPZ. BALB/c mice (4 mice per group) were immunized (iv) with *wt* or *spect (*−*)* ANKA *Pb*iSPZ (one dose of 1×10^5^ iSPZ). Seven (7) days later, PBL, liver and spleen cells of mice were isolated to determine frequency of *Pb*CSP-specific CD8+ T-cells by flow cytometry (FACScan). The CD8+ T-cells were double stained with PE-conjugated *Pb*CSP epitope tetramer and FITC-conjugated anti-mouse CD8b antibody. The naive group received neither *wt* nor *spect (*−*)* iSPZ. p-value compares statistically significant mean of CD8+ T-cell frequency between *wt* and *spect (*−*)* iSPZ-immunized groups.

Wild-type SPZs are known to be able to cross several cells (leaving a trail of the CSP behind) before infecting a single hepatocyte, unlike *spect (−)* SPZ that infect hepatocytes without cell traversal. It has been shown that both infected and traversed hepatocytes are able to process and present *Pb*SPZ CSP to primed CD8+ T-cells to induce IFN-γ secretion *in *vitro but only the infected hepatocytes were responsible for their own elimination [6], [20]. Similar experiments were then performed *in vivo*. Thus, it was expected that *spect (−)* SPZ would activate a lower number of the *Pb*CSP-specific CD8+ T-cells in the periphery and present a lower parasite load in the liver. RT-PCR clearly showed that the relative parasite DNA load in liver and spleen was significantly higher in mice receiving *wt* iSPZ compared to the *spect (−)* iSPZ parasite group (p = 0.026 and 0.008, respectively) (**Fig. 2a and b**). In addition, FACS analysis showed that the level of the *Pb*CSP-specific CD8+ T-cell response was significantly higher in the *wt* compared to the *spect (−)* group in PBL and spleen (p = 0.029) suggesting a role of cross-presenting DCs in these compartments **(** **Fig. 3** **)**. In contrast, in the liver, the frequency of CD8+ T-cells was similar (p  = 0.886) for the two kinds of iSPZ **(** **Fig. 3** **)** in spite of a lower parasite load for *spect (−)*. Both groups of mice were protected against live SPZ challenge **(** **Table 5** **).** Together, these data indicate that T-cell traversal by SPZ induces a higher level of immune response in PBL and spleen, but not in the liver, further supporting the key role of infected hepatocytes in antigen presentation and induction of protective immunity.

**Table 5:** Both *wt* and *spect (*−*) Pb*iSPZ immunization protect mice against SPZ challenge.

Immunization (number of *Pb*ISPZ)	Challenge with *Pb*SPZ	Mice protected/total challenged	% of protection
**Wild-type (1×10^5^)**	1×10^4^	5/5	**100**
**Spect (**−**) (1×10^5^)**	1×10^4^	5/5	**100**
**Naive (0)**	1×10^4^	0/5	**0**

One week after 1×10^5^ *Pb*iSPZ (*wt* or *spect (*−*)*) immunization, BALB/c mice were challenged with 1×10^4^ live ANKA *wt Pb*SPZ. Parasitemia was checked at 1 week and 2 weeks post challenge and mice were considered protected when they remained parasite negative 2 weeks after challenge.

## Discussion

Data presented in this manuscript provide further evidence for the role of *Plasmodium* infected hepatocytes in the stimulation of protective secondary CD8+ T-cells leading to the elimination of *P. berghei* pre-erythrocytic stages. In addition, they indicate that hepatocytes can indeed prime sporozoite-specific, protective naïve T-cells. While the antigen-presenting role of infected hepatocytes in the secondary CD8+ T-cell response seems to be accepted, the mode of priming naïve CD8+ T-cells is still, in our opinion, not yet established. With regard to the first point, the *in vivo* data presented here are fully consistent with our previous *in vitro* results [6], [20] and with *in vivo* data published by Zavala and collaborators [7] via bone marrow cell transfer experiments. In our case, we transferred either BALB/c infected hepatocytes or a CS specific T-cell clone or both to TAP-deficient H-2k^b^ mice. *Tap−/−*mice were chosen to bypass the possibility that antigen presentation is mediated by professional antigen presenting cells which might have ingested apoptotic infected hepatocytes [8] or live sporozoites possibly externally associated with BALB/c hepatocytes, and minimize host vs graft immune responses. In addition, we have determined protection as lack of infection in mice 14 days post-challenge, which represents a stringent, but the only significant standard for protection for pre-erythrocytic vaccines. Our results show that protection from infection is antigen-specific since mice are not protected if an irrelevant CD8+ T-cell clone is used. In addition, as observed *in vitro* [6], our results indicate that infected hepatocytes are directly killed by the antigen- specific T cells and not by a bystander effect through continuous secretion of IFN-γ or other lymphokines by the injected T-cell clones or host vs graft immune response since concomitant infection of TAP-deficient mice does not lead to protection after treatment with the CS specific T-cell clone and/or naïve BALB/c hepatocytes. These and the previous data [6], [7] clearly establish the central role of infected hepatocytes in the total clearance of *Plasmodium* infection *in vivo* once an immune response has been induced (secondary response). However, in our opinion, the role of hepatocytes in priming naïve, T-cells is not yet elucidated. Zavala and collaborators [7], [16] claim that peripheral dendritic cells prime CD8+ T-cells, while Leiriao *et* *al*. [8] suggest that apoptotic infected hepatocytes provide antigens to liver dendritic cells. In addition, these claims seem to be supported by the notion that hepatocytes act as tolerizing cells [27], [28]. On the other hand, given the large number of hepatocyte genes affected by sporozoites and salivary gland components, including some related to antigen processing and presentation and chemokine production it is not far fetched to hypothesize that infected hepatocytes become full-fledged antigen presenting cells upon infection [29], [30]. This would allow the activation of T-cells specific to sporozoite and late liver stage antigens (14). This would optimize the balance between infection and immune responses that parasites and hosts have developed through co-evolution. Evidence that the liver is central to obtaining an optimal immune response was provided early on by Renia *et al* [10] in which similar results as obtained here were presented, where immunization with non-parenchymal cells did not result in protection. Recent data by Epstein et *al*. [12] in which a better immune response was obtained by immunization of mice with irradiated sporozoites via iv than id or sc injection also point to the antigen-presenting role of the liver. In this study, we specifically target the role of infected hepatocytes in the immune response by immunizing (iv) mice with *spect (*−*)* iSPZ that infect hepatocytes without prior traversal. We show that, in spite of a significant decrease in parasite DNA load, the liver CSP-specific CD8+ T-cell response was similar to that in *wt* iSPZ-immunized groups. This and cyt c treatment of mice prior to immunization with iSPZs further support the key role of infected hepatocytes in T-cell priming to provide protective immune responses**.**

In conclusion, the data provided show that hepatocytes can indeed present *Plasmodium berghei* CSP epitopes to primed CD8+ T-cells and strongly suggest that they could also prime parasite-specific naïve T-cells to fully protect mice against a live parasite challenge. But in our opinion, formal proof of the role of hepatocytes in antigen presentation can only be obtained by isolating malaria parasite infected hepatocytes for *in vivo* and in *vitro* experiments.
